# Genome Sequences of *Gordonia terrae* Bacteriophages Phinally and Vivi2

**DOI:** 10.1128/genomeA.00599-16

**Published:** 2016-08-18

**Authors:** Welkin H. Pope, Kaitlyn C. Anderson, Charu Arora, Michael E. Bortz, George Burnet, David H. Conover, Gina M. D’Incau, Jonathan A. Ghobrial, Audrey L. Jonas, Emily J. Migdal, Nicole L. Rote, Brian A. German, Jill E. McDonnell, Nadia Mezghani, Claire E. Schafer, Paige K. Thompson, Megan C. Ulbrich, Victor J. Yu, Emily C. Furbee, Sarah R. Grubb, Marcie H. Warner, Matthew T. Montgomery, Rebecca A. Garlena, Daniel A. Russell, Deborah Jacobs-Sera, Graham F. Hatfull

**Affiliations:** Department of Biological Sciences, University of Pittsburgh, Pittsburgh, Pennsylvania, USA

## Abstract

Bacteriophages Phinally and Vivi2 were isolated from soil from Pittsburgh, Pennsylvania, USA, using host *Gordonia terrae* 3612. The Phinally and Vivi2 genomes are 59,265 bp and 59,337 bp, respectively, and share sequence similarity with each other and with GTE6. Fewer than 25% of the 87 to 89 putative genes have predictable functions.

## GENOME ANNOUNCEMENT

*Gordonia* spp. are common soil inhabitants, and the genus contains a diverse array of species. Relatively few *Gordonia* phages have been isolated and genomically characterized, although those that have display various different genomic structures (1–5). The Science Education Alliance Phage Hunters Advancing Genomics and Evolutionary Science (SEA-PHAGES) program presents an opportunity to isolate and characterize additional phages of *Gordonia* hosts in order to understand their diversity and evolution (6, 7).

*Gordonia* phages Phinally and Vivi2 were isolated from enrichment cultures of soil samples found in Pittsburgh, Pennsylvania, USA, using *Gordonia terrae* 3612 as a host. Electron microscopy reveals that both have siphoviral morphologies with 290 to 300 nm flexible tails, as well as isometric heads. Following plaque purification and amplification, DNA was extracted and sequenced using an Illumina MiSeq with single-end 140-bp reads. Reads were assembled using Newbler into one major contig each of 59,265 bp and 59,337 bp with 285-fold and 673-fold coverages, respectively. Assembly indicated that the genomes are circularly permuted with 68.4% (Phinally) and 67.1% (Vivi2) G+C% contents. Base one was assigned to the first nucleotide of the predicted gene immediately upstream of and slightly overlapping the predicted terminase in each genome. Eighty-seven and 89 protein-coding genes were predicted for Phinally and Vivi2, respectively, using Glimmer (8), GeneMark (9), DNA Master (http://cobamide2.bio.pitt.edu), and Phamerator (10). Functions were assigned to predicted virion structure and assembly genes, RecE/T, endolysin, DNA methylase, and exonuclease genes using BLASTp (11) and HHpred (12) and the publically available databases GenBank, the Protein DataBase, and pFamA. No tRNA genes or putative integrase or repressor genes associated with temperate lifestyles were identified. All genes are transcribed rightward in both genomes with the exception of leftward-transcribed HNH endonuclease genes in Vivi2.

Vivi2 and Phinally have moderate nucleotide sequence similarity with each other and to phage GTE6 (5). The degree of similarity is low (~70%) and the genome matches span only about 50% of the genome lengths. In general, the matching segments are dispersed throughout the genomes, and the matching distances are similar between the three phages. Phinally, Vivi2, and GTE6 differ somewhat in some of their minor tail proteins, perhaps reflecting distinct host preferences, and we note that GTE6 infects several strains of *G. terrae*, *G. malaquae*, and *G. hydrophobica* (5).

Phinally and Vivi2 both code for related N-6 adenine specific DNA methylases but at distinct genomic locations, and Vivi2 has a cytosine C-5 specific DNA methylase gene between the terminase and portal genes. All of these are absent from GTE6, although there are homologs in cluster A, cluster F, and cluster I mycobacteriophages and thus may be relatively recent additions to the Phinally and Vivi2 genomes. We have not been able to identify restriction endonuclease genes associated with the methylases. All three genomes encode a RecET-like recombination system and a putative RuvC-like Holliday junction resolvase. However, their substantial differences in gene content are reflected in their combined contribution of 100 genes that have no close relatives among the 150,000 genes of actinobacteriophages sequenced to date (http://phagesdb.org).

### Accession number(s).

The Phinally and Vivi2 genome annotations are available from GenBank under the accession numbers KU963253 and KU963250.
